# Omega-3 LC-PUFA consumption is now recommended for women of childbearing age and during pregnancy to protect against preterm and early preterm birth: implementing this recommendation in a sustainable manner

**DOI:** 10.3389/fnut.2024.1502866

**Published:** 2024-11-29

**Authors:** Ella J. Baker, Philip C. Calder, Alex J. Kermack, Jonathan E. Brown, Moriam Mustapha, Ellen Kitson-Reynolds, Josephine J. Garvey

**Affiliations:** ^1^School of Human Development and Health, Faculty of Medicine, University of Southampton, Southampton, United Kingdom; ^2^NIHR Southampton Biomedical Research Centre, University Hospital Southampton NHS Foundation Trust and University of Southampton, Southampton, United Kingdom; ^3^Department of Obstetrics and Gynaecology, Princess Anne Hospital, University Hospital Southampton NHS Foundation Trust, Southampton, United Kingdom; ^4^School of Medicine, Faculty of Health and Medical Sciences, University of Surrey, Guildford, United Kingdom; ^5^Department of Nutrition, Food and Exercise, School of Biosciences, Faculty of Health and Medical Sciences, University of Surrey, Guildford, United Kingdom; ^6^London Neonatal Operational Delivery Network, London, United Kingdom; ^7^School of Health Sciences, Faculty of Environmental and Life Sciences, University of Southampton, Southampton, United Kingdom; ^8^Consultant, Amsterdam, Netherlands

**Keywords:** omega 3 (n-3) polyunsaturated fatty acids, pregnancy, preterm (birth), sustainable omega 3s, maternal health

## Abstract

Preterm birth (delivery prior to 37 weeks) appears to be rising globally, increasing the risk of a myriad of down-stream disorders which affect families, their offspring and society, including increased morbidity, mortality and economic costs. Strategies for prevention of preterm birth have therefore become a priority among healthcare providers. One proposed strategy is increased consumption of Omega-3 long-chain polyunsaturated fatty acids (LC-PUFAs), particularly docosahexaenoic acid (DHA) (from food or supplements) in women of childbearing age and during pregnancy. It is hypothesized that Omega-3 LC-PUFAs, through several different actions, reduce the risk of early onset labor or lengthen gestation. An expert group, acting on behalf of several relevant organizations, recently published guidance based on compelling trial evidence for increased Omega-3 LC-PUFA intake to protect women of childbearing age and during pregnancy from preterm birth (PTB) and early preterm birth (ePTB). Here, we consider how this guidance can be achieved in a sustainable manner. We present data on suitable, efficacious alternatives to fish as a source of Omega-3 LC-PUFAs, so that while aiming to protect families and society against PTB and ePTB there is no increased burden on other species on our vulnerable planet. Finally, how the guidance can be implemented in practice is discussed, with consideration for those most at risk and effective ways of communicating this important message.

## Introduction

### Prevalence/incidence of preterm birth

Preterm (premature) birth (PTB) is defined as birth before 37 weeks gestation. The United Nations (UN) estimates that globally 13.4 million babies were born preterm in 2020, with nearly one million dying from preterm complications (1). This represents about 1 in 10 babies globally being born prematurely (1). Reports from Europe indicate that between 4.0 and 8.2% of all singleton live births are preterm, with significantly higher rates (up to 74.8%) in multiple pregnancies, although this may be artificially raised due to iatrogenic delivery of some multiple pregnancies, particularly monochorionic, prior to 37 weeks in accordance with guidelines (2). It has also been suggested that these numbers may be increasing in some regions (3). The Centers for Disease Control and Prevention (CDC) reported that the percentage of newborns delivered preterm in the United States (US) rose from 10.09% in 2020 to 10.49% in 2021 (4) with some groups, such as African Americans, experiencing increases of up to 14.4% (4). Globally, most (around 65%) PTBs occurred in sub-Saharan Africa and southern Asia with the greatest incidence rates seen in Bangladesh, Malawi and Pakistan (1). Around two-thirds of PTBs occur spontaneously in otherwise healthy women (5), while medically-induced or iatrogenic PTB also exists when in the best interest of the mother or baby, for example, in conditions such as preeclampsia. It is reported that PTB, and especially early PTB (ePTB: <34 weeks gestation), accounts for 85% of all perinatal complications (6).

### The ripple effect of PTB and ePTB on infant/childhood well-being

Children born preterm have an increased risk of a myriad of disorders both physical and neurological, many of which can be lifelong, and these can exert substantial social, psychological and economic challenges on families and society (6). The annual economic cost of prematurity in the US was estimated to be as high as $25.2 billion (national aggregate costs in 2016) when lost productivity costs were also included (7), and increased by 4% from 2004 to 2016 when adjusted for inflation (7). Additional downstream costs related to illness resulting from PTB or ePTB (e.g., life-long management of conditions such as cerebral palsy) further add to this cost (8).

Studies suggest that increased survival of preterm, and especially early preterm, infants raises concerns due to the numbers who will go on to develop complex morbidities such as necrotising enterocolitis, broncho-pulmonary dysplasia, severe visual and hearing impairments, cerebral palsy, and cognitive developmental delay (9). In addition, there is an increased risk of developing non-communicable diseases (NCDs) such as cardiovascular disease, metabolic syndrome and obesity in later life (10, 11). These chronic morbidities place further challenges on the healthcare system.

Strategies for prevention of PTB and ePTB have therefore become a priority. In the UK for example, Saving Babies Lives version 3, The Department of Health’s “Safer Maternity Care” report, extended the “Maternity 4 Safety Ambition” to include reducing preterm births from 8 to 6% (12). Multiple strategies have been proposed to do this, including implementing PTB leads and clinics in each healthcare organization, promoting smoking cessation and preventative methods such as cervical length scanning, progesterone use and cervical cerclage for those at high risk. Increasing the consumption of omega-3 long chain polyunsaturated fatty acids (LC-PUFAs) in women of childbearing age and during pregnancy has also been proposed as a preventative strategy (2).

### Omega-3 long chain polyunsaturated fatty acids

Omega-3 fatty acids are a family of fatty acids that are considered essential nutrients for health. The parent fatty acid of this family is *α*-linolenic acid (α-LA) which cannot be produced by the human body due to the inherent inability of humans to desaturate oleic acid between the centrally-located double bond and its methyl terminus and therefore *α*-LA must be provided by the diet. One of the many important functions of α-LA is providing the LC-PUFAs docosahexaenoic acid (DHA) and eicosapentaenoic (EPA). DHA and EPA are synthesized from α-LA via desaturation and elongation, initially involving the sequential action of the enzymes delta 6 desaturase, elongase and delta 5 desaturase to produce EPA and then further desaturation and elongation to produce DHA. However, conversion from *α*-LA to the LC-PUFAs, especially to DHA, is considered to be relatively low (13). This places an emphasis on the need to consume preformed EPA and DHA in the diet. The richest dietary source of EPA and DHA is seafood, especially oily fish such as salmon, mackerel, sardines, and tuna. However, some freshwater fish such as trout are also rich in EPA and DHA.

Pregnancy imparts special nutritional needs on the mother and the fetus, and almost all nutrient needs are higher than in the non-pregnant state (14). The growing fetus has a requirement for preformed LC-PUFAs, such as DHA, which are supplied preferentially by placental transfer (15). Placental synthesis of LC-PUFAs is considered to be low (with limited activity of the requisite enzymes) and unlikely to meet fetal demands (16). Therefore, the mothers’ stores (accumulated pre-pregnancy) and dietary intake (during pregnancy) represent the main supply of DHA to the fetus (16). Burdge et al. (17) reported that infants born to vegetarian mothers had lower status of DHA compared to those born to omnivores, possibly signifying the absence of preformed DHA in the mothers’ diet. Recent interest has focused on the importance of these omega-3 LC-PUFAs, and especially DHA, in protecting against pregnancy disorders, such as PTB and ePTB. Currently, in many countries, including the United Kingdom, the benefits of consuming omega-3 LC-PUFAs in terms of protection against PTB and ePTB are not discussed with pregnant women.

### The hypothesis—omega-3 LC-PUFAs protect against PTB

The hypothesis for protection from PTB by omega-3 LC-PUFAs was developed over 30 years ago based on data from the Faroe Islands (18). The authors proposed that omega-3 LC-PUFAs increased birthweight by prolonging gestation through interference with uterine prostaglandin (PG) production, inhibiting the production of the 2-series PGs involved in uterine contractions and promoting production of the 3-series PGs involved in cervical ripening (18). Omega-3 LC-PUFAs were also thought to be protective against placentation disorders (19). The proposed mechanism for this is that omega-3 LC-PUFAs, especially DHA, enhance the invasion of trophoblasts and the transformation of spiral arteries into larger vessels, reducing the risk of ischemia (19). Carvajal proposed that failure of normal placentation generates a series of clinical abnormalities called “deep placentation disorders” including preeclampsia, fetal growth restriction, preterm labor, premature rupture of membranes, *in utero* fetal death and placental abruption (19). Omega-3 LC-PUFAs also appear to support the relaxation of the myometrium, averting the early onset of labor through “antiarrhythmic” effects on the myometrium (20). Furthermore, omega-3 LC-PUFAs seem to limit oxidative damage and enhance reactive oxygen species (ROS) scavenging, as well as reducing inflammatory responses implicated in some PTBs (16). Finally, omega-3 LC-PUFAs have been associated with the regulation of oxytocin signaling which may prolong gestation (21). Consequently, omega-3 LC-PUFAs appear to protect against both spontaneous PTB as well as the need for medically-induced PTB, for example as a result of preeclampsia.

Numerous studies have been performed over the years demonstrating the benefits of omega-3 LC-PUFA consumption in the protection against PTB and ePTB, as well as many other additional benefits (22–24). These data have been eloquently summarized in the recently, supporting evidence-based guidance for intake of omega-3 LC-PUFAs in order to safeguard women of childbearing age, as well as pregnant women, against PTB and ePTB (2).

## The recent guidance

Clinical practice guidance was recently developed by a group of international experts from several medical and scientific organizations (2), concluding that in order to reduce the risk of PTB and ePTB “*women of childbearing age should obtain a supply of at least 250 mg/d of docosahexaenoic acid (DHA) +* eicosapentaenoic *(EPA), from diet or supplements. While, during pregnancy they should receive an additional ≥ 100 to 200 mg/d of DHA*.” In addition, the guidance proposes that pregnant women “*with a low status,* i.e.*, low DHA intake and/or low DHA blood levels*,” who have an increased risk of PTB and ePTB birth should receive “*approximately 600 to 1,000 mg/d DHA + EPA respectively, or DHA alone*.” This guidance is based on data from numerous clinical trials, showing that at this dosage there was a significant reduction of PTB and ePTB (6, 20, 25–31). Furthermore, the guidance proposes that “*this additional supply should preferably begin in the second trimester of pregnancy (not later than approximately 20 weeks’ gestation) and continue until approximately 37 weeks’ gestation or until childbirth if before 37 weeks’ gestation*.” The identification of women with inadequate omega-3 intakes might, the authors suggest, be achievable by using a set of standardized questions on intake. DHA measurement in blood was proposed as another option to identify women with low status, although the authors concluded that standardization of laboratory methods and appropriate cutoff values were still needed (2). Finally, it is suggested that “*information should be provided to*” women of childbearing age and pregnant women as well as their partners “*on how to achieve an appropriate intake*” of DHA or DHA + EPA.

These guidelines are based on compelling scientific evidence from randomized clinical trials (going back almost 25 years) demonstrating the benefits of omega-3 LC-PUFAs for the protection against PTB and ePTB; and constitute a formal consensus by experts in the field. As a follow-on from the guidelines, the European Board and College of Obstetrics and Gynaecology (EBCOG) came with a position statement where they concluded that the consumption of foods rich in omega-3 LC PUFAs, or supplements, was associated with a 11 and 42% risk reduction of early and late preterm births, respectively (32).

The aim of this review is to (a) discuss how to apply these important guidelines in practice and (b) consider how to achieve them in a manner that is sustainable for the planet.

### Implementing the guidelines: practical considerations

Despite the undoubted importance of this new guidance, several considerations remain in terms of their implementation in practice. These include consideration for methods (i.e., how to get the correct message across knowing that current recommendations for omega-3 LC PUFA intake in the general population are not met), who the advice should be given to (all women, those with low omega-3 LC-PUFA status, those at high risk of PTB and, if advice is based on status, how to assess this in a cost-effective way), who should be responsible for giving the advice (data suggest pregnant women favor physicians who themselves need education), and the long-term viability of the guidance (thinking about sustainability/the planet).

### Which groups are most at risk?

Women at risk of having a PTB or ePTB include those aged <20 years (33), those living with obesity (34), those with advanced age and complications during pregnancy (33, 35) and potentially those with a low omega-3 LC-PUFA status (6), especially DHA. In addition, when there is an economic crisis, which in some places worsened following the COVID-19 pandemic, pregnant women might be less able to source a healthy diet and/or acquire supplements (36). If money is an issue, women often prioritize their family before themselves, especially when they have other competing demands (37, 38).

Meanwhile, the risk of having low DHA status may be greater in women with pregnancy complications such as diabetes or preeclampsia, as well as obesity (39), and these conditions have been shown to have a negative effect on delta-6-desaturase activity (40–42), so reducing endogenous synthesis of omega-3 LC-PUFAs, as well as placental transfer of LC-PUFAs (39). Makrides et al. (25) reported that 80% of Australian women consumed some DHA during pregnancy, averaging ~150 mg/day. In the US, Gustafson et al. (39) reported that many women were taking prenatal DHA supplements with a mean intake of 104 mg/day. However, diet provided relatively small amounts of DHA, ~65 mg/day in the US (39).

Having a low omega-3 LC-PUFA status has been defined as red blood cell (RBC) phospholipid DHA (RBC-DHA) < 6% of total fatty acids (2). Simmonds et al. (24) found that women with a higher total omega-3 status (in RBCs) in early pregnancy had a lower risk of ePTB. Meanwhile Makrides et al. (25) reported that when DHA status was <6% at baseline then 1,000 mg of DHA had the biggest impact on ePTB protection. Women at risk of PTB include women with diabetes, those with a previous PTB or mid-trimester loss, previous preterm pre-labor rupture of membranes at less than 34 weeks, previous shortened cervix requiring cervical cerclage, previous uterine variant, previous birth by cesarean section at full dilatation, or previous cervical surgery including a Large Loop Excision of the Transformation Zone (LLETZ) procedure where >15 mm was removed or > 1 LLETZ.

### Achieving these guidelines in a sustainable manner

It is well described that there are not enough fish in the oceans and rivers to sustainably support recommended intakes of omega-3 LC PUFAs (43). There were around 1.8 billion women of reproductive/childbearing age globally in 2019 and this is expected to increase to 2 billion by 2025 (44). If the omega-3 LC-PUFA guidelines were followed by just these women, the environmental impact would be huge. If each of these women were to consume at least 250 mg/d of DHA + EPA from fatty fish, this would equate to around 84 g of (skipjack, cooked) tuna/day or 29 g of (sockeye, cooked) salmon/day, or around 2–3 servings/week. When applied globally this would have a huge negative environmental impact and is not sustainable. The oceans and wild fisheries are already operating at full capacity causing concerns about the future quality and availability of food. The European Commission developed a common “*Fisheries and oceans pact towards sustainable, science-based, innovative and inclusive fisheries management*” which aims to focus attention on long-term sustainability for fisheries and aquaculture (45). This report states that fishing activities continue to adversely affect marine ecosystems, particularly through seabed disturbance, bycatch of sensitive species and effects on marine food webs (45). At the same time, the poor status of marine ecosystems is a direct threat to the sustainability of fisheries and aquaculture. Plastic, micro plastic and other pollutants from human activities at sea and on land (e.g., agriculture, fisheries, industry, shipping, waste waters) also have a negative impact on marine ecosystems and, consequently, on fisheries and aquaculture activities. Other aspects to consider are that the richest sources of omega-3 LC-PUFAs are cold water sea species, that these fish accumulate fatty acids via the food chain and that current sources of omega-3 LC-PUFAs are mostly fish-sourced. Raising ocean temperatures are likely to drive organisms lower down the food chain away from producing LC-PUFAs toward producing shorter chain and/or more saturated fatty acids which would reduce the entry of omega-3 LC-PUFAs into the food chain (46).

## Are there alternatives to fish as sources of omega-3 LC-PUFAs?

### Algae and algal oils

In recent years non-fish sourced DHA + EPA, mostly derived from algae, have become available. These are a sustainable alternative to consuming fish or using fish as the source of oil for supplements and help protect the planet by avoiding overfishing to meet human needs. Furthermore, algal oils are suitable for vegetarian and vegan consumers as supplemental sources of omega-3 LC-PUFAs. Heterotrophic microalgal species such as *Schizochytrium, Aurantiochytrium*, *Thraustochytrium*, and *Crypthecodinium cohnii* are essential producers of DHA (47). Oils produced from algae typically contain more DHA than fish oil (20–55% of fatty acids) with some EPA (Table 1) (47). Algal oils have been used in the infant formula industry for many years, providing DHA at a concentration to match that present in human breast milk, and evidencing their safety. Furthermore, algal oils have been shown to increase EPA and DHA status in blood cells in adults just like fish oil does (48). Algal oils providing DHA have been shown to raise DHA status in pregnant women (49) and such supplements have been used in many of the trials investigating omega-3 LC-PUFAs and risk of PTB.

**Table 1:** Typical omega-3 fatty acid composition of commonly available plant and algal oils.

Oil source	Omega-3 fatty acids (% total fatty acids by weight)
Soybean	α-LA (5–10%)
Canola (rapeseed)	α-LA (6–14%)
Flaxseed	α-LA (40–60%)
Echium	α-LA (28.4%) and SDA (12.5%)
Ahiflower	α-LA (45%) and SDA (20%)
GM soybean	α-LA (9–12%) and SDA (15–30%)
**Algae**	
Heterotrophic species	Predominately DHA (20–55%)
Photosynthetic species	Predominantly EPA (36–46%)

α-LA, alpha-linolenic acid; DHA, docosahexaenoic acid; EPA, eicosapentaenoic acid and SDA, stearidonic acid. Data taken from Nandasiri et al. (60), Wan Ghazali et al. (61), Goyal et al. (62), Lefort et al. (63), Sijtsma and de Swaaf (64), Guil-Guerrero (65), and Deckelbaum and Torrejon (66).

Therefore, evidence supports that algal oils are a sustainable source of omega-3 LC-PUFAs for pregnant women. However, these alternative omega-3 LC-PUFA sources are not always available and can be expensive to purchase, adding to the already high cost of food which can be a challenge for some populations. For example, there are combinations of EPA and DHA from algae on the market with levels ranging from 100 to 165 mg EPA and 300–330 mg DHA per g of oil, but these supplements are expensive. To meet the most modest of recommendations, one capsule per day would be needed but to meet the less conservative recommendations two or even three capsules daily would be needed.

### Oils from genetically-modified plants

Genetically-modified plants may represent a future sustainable source of omega-3 LC-PUFAs. Terrestrial plants do not usually produce EPA and DHA. Two oilseed crops, *Brassica napus* (rapeseed, also known as canola) and *Camelina sativa* have been genetically modified to produce EPA and DHA. Genetically modified canola lines have been developed that contain modest EPA and low DHA (7 and 1% of fatty acids, respectively) or modest DHA and low EPA (10 and 1%, respectively) (50). Several genetically modified Camelina lines have been generated, some producing high EPA (up to 30% of fatty acids) and relatively little DHA, some high DHA and modest EPA (12 and 3% of fatty acids, respectively), and others producing high amounts of both (e.g., EPA 12% and DHA 14% or EPA 11% and DHA 8%) (51). Therefore, the EPA and DHA contents in the oil from some camelina lines are similar to the amounts in standard fish oils. Oils from genetically modified canola and camelina lines have been studied in humans and they behave similarly to fish oils (52). Together these studies highlight the potential for oil from genetically modified terrestrial plants to be a sustainable alternative to marine-sourced EPA and DHA. Currently, there are no data to support the use of genetically modified oils in pregnancy, and there are several barriers to the use of oils from genetically modified plants, including regulatory issues, cost, and public acceptability. The challenges posed by genetic modification of plants to enhance their characteristics have been discussed elsewhere (53). Women may be more reluctant to accept genetically modified food than men (54) and this reluctance may be exaggerated by pregnancy. There needs to be an evidence-based strategy to communicate the likely benefits of oils from genetically modified plants that are rich in EPA and DHA.

### Non-genetically modified plant oils

Non-genetically modified plant-sourced omega-3 fatty acids may also provide a sustainable alternative to omega-3 LC-PUFAs, relying upon biological activity in their own right or acting as precursors for biosynthesis of EPA and DHA (13). Omega-3 PUFAs derived from plants include *α*-LA and stearidonic acid (SDA). α-LA is found in green leaves, some seeds and nuts, and in some plant oils including soybean and canola oils. Flaxseeds and flaxseed oil are a very rich source of *α*-LA, which contributes about 55% of the fatty acids present. Chia seeds are also rich in *α*-LA (60% of fatty acids). α-LA has been shown to have some bioactivity in its own right, including reduction of total and low-density lipoprotein cholesterol (55) and production of bioactive oxylipins (56). Furthermore, *α*-LA is a precursor for omega-3 LC-PUFA synthesis and has been shown to increase EPA concentrations (13); however, evidence suggests conversion to DHA is limited. Increased consumption of α-LA may provide a sufficient increase in EPA levels, however, the required increases in DHA concentrations may not be met by α-LA (13).

SDA is found in some plant sources including seeds from members of the Boraginaceae family of plants, including the genera Borago (borage), Echium (e.g., Viper’s bugloss), and Buglossoides (e.g., Corn gromwell). Some plant seed oils naturally contain SDA including those from *Echium plantagineum*, where SDA makes up about 12% of fatty acids, and from *Buglossoides arvensis*, known as Ahiflower, where SDA makes up about 20% of fatty acids (Table 1). These oils also contain *α*-LA (~33% and ~45% respectively) (Table 1). There is also oil from genetically modified soybean that contains SDA (15–30%) (Table 1). SDA has been shown to be a better precursor for EPA synthesis than α-LA. For example, James et al. (57) demonstrated that 1.5 g SDA per day for 6 weeks increased plasma and red blood cell EPA to a greater extent (~5-fold) than 1.5 g α-LA per day. Older studies with pure SDA or Echium oil reported similar but weaker effects than those reported for EPA and DHA (58), suggesting bioactivity of SDA is largely through conversion to EPA. More recent studies examining the effects of different doses of Ahilfower oil showed dose-dependent increases in SDA, its derivative eicosatetraenoic acid and EPA in plasma and in blood mononuclear cells, but no increase in DHA (59). Table 1 presents the typical omega-3 fatty acid content found in commonly available plant oils.

## Communicating the message

To date, the message that omega-3 LC-PUFA consumption is protective against PTB and ePTB has not effectively reached key target populations. In order to achieve this, national and international organizations need to work together to come up with a consistent message on amounts and (safe, sustainable) sources of omega-3 LC-PUFAs needed for protection. Secondly, experts and organizations need to consider how to best disseminate agreed messages, considering educational tool needs. Consideration should be given to how to capture and engage specific target populations, for example, capturing women of childbearing age may pose an even greater challenge compared to pregnant women, who typically interact with midwives or obstetricians (especially in high-income countries), from whom they could receive advice. One way might be through school education platforms or government actions, similar to the folic acid campaign (see Table 2). Finally, advice could also be provided at specialist antenatal clinics in the form of e-learning modules, for example, to upskill those who interact with pregnant women, attended by those most at risk of having a PTB.

**Table 2:** Potential ways to disseminate omega-3 LC-PUFA messaging at national and local levels.

At national/public health level: Public education on increased consumption of omega-3 LC-PUFAs from a variety of sustainable sources Legislation on the level of omega-3 LC-PUFAs required in antenatal supplements sold over-the-counter and on prescription Education and training for healthcare professionals who provide first-contact care to pregnant women
At local level: Education for pregnant women on safe levels of omega-3 LC-PUFA consumption from a variety of sustainable source Prescription of omega-3 LC-PUFA supplements for pregnant women identified at high risk of preterm birth

### Summary

Recent international guidance based on compelling trial evidence favors consumption of omega-3 LC-PUFAs (EPA + DHA) to protect women of childbearing age and during pregnancy from PTB and ePTB. Fish is currently the major source of omega-3 LC-PUFAs either as a food or as the origin of most EPA and DHA supplements (“fish oils”). Here we discuss efficacious sustainable alternatives to fish/fish oils as a source of omega-3 LC-PUFAs, such as algal oils. These are safe and appear to be the most effective non-animal-based alternatives to fish in increasing EPA and DHA levels in humans; one barrier to their use though is that current sources are expensive. An alternative, terrestrial plant-based option that may be less expensive but that needs further exploration is oils that contain EPA and DHA from GM plants, although there are currently challenges to using these oils for direct human consumption. Another alternative is plant oils naturally rich in *α*-LA and SDA which are precursors to EPA. How to communicate the message about omega-3 LC-PUFAs to target populations needs consideration, but we propose practical ideas on this.
